# Evaluation of functional annotation-informed and ancestry-specific polygenic risk scores for ischemic stroke

**DOI:** 10.3389/fbinf.2026.1875388

**Published:** 2026-09-10

**Authors:** Nicole D. Armstrong, Vinodh Srinivasasainagendra, Amit Patki, Lavanya Pilla, Chad Aldridge, Alana C. Jones, Ulrich Broeckel, Ethan M. Lange, Leslie A. Lange, Pankaj Arora, Nita A. Limdi, Hirotaka Iwaki, Lana Sargent, Joohyun Kim, Maggie C. Y. Ng, Josep M. Mercader, Keith L. Keene, Bradford B. Worrall, Hemant K. Tiwari, Marguerite R. Irvin

**Affiliations:** 1 Department of Epidemiology, University of Alabama at Birmingham, Birmingham, AL, United States; 2 Department of Biostatistics, University of Alabama at Birmingham, Birmingham, AL, United States; 3 Department of Neurology, University of Virginia, Charlottesville, VA, United States; 4 Department of Pediatrics and Genomics Sciences and Precision Medicine Center, Medical College of Wisconsin, Milwaukee, WI, United States; 5 Department of Medicine, Anschutz Medical Campus, University of Colorado, Aurora, CO, United States; 6 Division of Cardiovascular Disease, University of Alabama at Birmingham, Birmingham, AL, United States; 7 Department of Neurology, University of Alabama at Birmingham, Birmingham, AL, United States; 8 Center for Alzheimer’s and Related Dementias, National Institute on Aging, Bethesda, MD, United States; 9 DataTecnica LLC, Washington, DC, United States; 10 School of Nursing, Virginia Commonwealth University, Richmond, VA, United States; 11 Division of Genetic Medicine, Vanderbilt Genetics Institute, Vanderbilt University Medical Center, Nashville, TN, United States; 12 Programs in Metabolism and Medical & Population Genetics, Broad Institute of Harvard and MIT, Cambridge, MA, United States; 13 Center for Genomic Medicine, Massachusetts General Hospital, Boston, MA, United States; 14 Diabetes Unit, Massachusetts General Hospital, Boston, MA, United States; 15 Department of Medicine, Harvard Medical School, Boston, MA, United States; 16 Novo Nordisk Foundation Center for Genomic Mechanisms of Disease, Broad Institute of MIT and Harvard, Cambridge, MA, United States; 17 Department of Biology, East Carolina University, Greenville, NC, United States; 18 Center for Health Disparities, East Carolina University, Greenville, NC, United States; 19 Department of Public Health Sciences, University of Virginia, Charlottesville, VA, United States

**Keywords:** functional annotation, genetic epidemiology, ischemic stroke, polygenic risk score, precision medicine

## Abstract

**Introduction:**

Ischemic stroke (IS) is a leading cause of morbidity and mortality, and predicting events remains challenging. Polygenic risk scores (PRSs) aggregate genetic variants, but performance varies across ancestries and remains modest, particularly in non-European populations.

**Methods:**

We compared Bayesian PRS methods incorporating functional annotations and expanded linkage disequilibrium reference panels for IS prediction. PRSs were generated using PRS-CS with HapMap3 and TagIt panels, and SBayesRC integrating functional priors. Scores were optimized in 1,403 European ancestry (EA) and 6,342 African ancestry (AA) participants from the Reasons for Geographic and Racial Differences in Stroke (REGARDS) study and independently validated in All of Us participants (78,155 EA and 29,594 AA participants), a REGARDS holdout cohort (11,459 EA participants), and the Genetics of Hypertension Associated Treatments (GenHAT) study (6,908 AA participants). Predictive performance was evaluated using logistic regression adjusted for age, sex, the top 10 genetic principal components, and antihypertensive treatment assignment where applicable. Performance was assessed using odds ratios per standard deviation (OR/SD), liability-scale R^2^, and area under the curve (AUC).

**Results:**

Among EA participants, SBayesRC demonstrated the largest effect size and liability R^2^ in the REGARDS holdout validation cohort (OR/SD = 1.24, 95% CI 1.12–1.36, R^2^ = 2.42%). PRS-CS using the expanded TagIt panel (OR/SD = 1.11, 95% CI 1.00–1.22) showed numerically stronger performance than the HapMap3 panel (OR/SD = 1.09, 95% CI 0.98–1.20). Performance was consistent across the All of Us EA cohort, with OR/SD ranging from 1.10 to 1.16 across methods. Among AA participants, associations and predictive metrics were attenuated, with no significant associations observed in All of Us. A modest association was observed for PRS-CS-TagIt in GenHAT (for an all-stroke outcome). PRSs modestly improved discrimination beyond covariate models among EA participants (AUCs up to 66.6%), but showed little to no improvement among AA participants (AUCs 62.7%–66.7%). High PRS percentile groups (e.g., top 2% versus 98%) showed modest stroke enrichment with high specificity and limited sensitivity.

**Conclusion:**

Functional annotation-informed PRSs and expanded reference panels modestly improved prediction, particularly among EA participants. Limited performance among AA participants underscores the need for larger, more diverse genomic datasets and integrative models incorporating genetic, clinical, lifestyle, and social determinants of health.

## Introduction

Stroke is a leading cause of death and disability globally, resulting in more than 7 million deaths per year (Palaniappan et al., 2026). Beyond mortality, stroke imposes a substantial public health burden (Taylor et al., 1996) through prolonged hospitalization, long-term rehabilitation, loss of independence, and decreased quality of life for survivors and their families (Godwin et al., 2013; Alhusayni and Alzahrani, 2025; Strilciuc et al., 2021; Donkor, 2018). Despite advances in prevention and acute treatment, predicting who will experience a stroke remains challenging. Improved risk prediction tools that can identify high-risk individuals earlier could enable targeted prevention strategies, optimize clinical management, and ultimately reduce the individual and societal burden of stroke across diverse populations.

While well-established behavioral and clinical risk factors, such as body mass index, hypertension, type 2 diabetes, and cigarette smoking, play a major role in stroke risk (Chen et al., 2014), there is compelling evidence for a heritable component. Twin and family studies indicate that ischemic stroke has heritability estimates ranging from 16% to 40%, depending on the subtype and population studied (Bevan et al., 2012; Traylor et al., 2017) Recent genome-wide association studies (GWAS), including large consortia efforts such as MEGASTROKE (Malik et al., 2018), GIGASTROKE (Mishra et al., 2022), and COMPASS (Keene et al., 2020), have identified dozens of loci associated with ischemic stroke (IS) and its subsequent subtypes. However, the majority of these variants individually confer modest effect sizes, and their translational potential for clinical risk prediction remains limited.

Polygenic risk scores (PRSs) offer a promising framework to translate GWAS findings into individualized risk prediction by aggregating the effects of thousands to millions of common variants across the genome (Lewis and Vassos, 2017). Several statistical methods have been developed to improve PRS performance, with Bayesian approaches, such as PRS-CS, demonstrating improved accuracy by incorporating genome-wide variants and applying continuous shrinkage priors to variant effect sizes (Ge et al., 2019). However, PRS performance varies significantly across ancestral populations, in part due to differences in linkage disequilibrium (LD) patterns, allele frequencies, and underrepresentation of non-European ancestry populations in GWAS summary statistics (Martin et al., 2017). In stroke specifically, PRS research remains comparatively sparse, with approximately 30 scores overall in the Polygenic Score (PGS) catalog (Lambert et al., 2021; Lambert et al., 2024), in contrast to over 70 scores developed for coronary artery disease. Furthermore, recent stroke PRS studies, such as those by Abraham et al*.* (Abraham et al., 2019) and Mishra et al. (Mishra et al., 2022) have shown that current scores, as well as composite meta-scores, offer only modest incremental value beyond clinical risk factors, particularly in non-European populations.

To address this gap, we evaluated whether incorporating functional annotations and expanded ancestry-specific LD reference panels improves IS PRS performance. We hypothesized that leveraging functional priors would enhance the prioritization of putatively causal variants and improve PRS performance, particularly in non-European populations. We generated and compared two Bayesian PRS approaches: (i) PRS-CS (Ge et al., 2019) using the standard HapMap3 (HM3) LD reference panels and with an expanded, multi-ancestry LD reference panel (TagIt) developed by the D-PRIME consortium (Huerta-Chagoya et al., 2026), and (ii) SBayesRC (Zheng et al., 2024), which integrates functional annotations. These methods were assessed using individual-level data in 1,403 self-reported European ancestry (EA) and 6,342 self-reported African ancestry (AA) participants from the Reasons for Geographic and Racial Differences in Stroke (REGARDS) study. Validation was performed in independent cohorts, including 78,155 (898 ischemic stroke cases) EA participants from the All of Us Program, 11,459 (444 IS cases) holdout EA REGARDS participants, 29,594 (311 IS cases) AA All of Us participants, and 6,908 (366 any stroke) AA participants from the Genetics of Hypertension Associated Treatments (GenHAT) study. For contextual benchmarking, we also evaluated two previously published stroke PRSs (Abraham et al. (Abraham et al., 2019) and Mishra et al. (Mishra et al., 2022)).

## Methods

### Study populations

#### The Reasons for Geographic and Racial Differences in Stroke (REGARDS) study

REGARDS is a national, population-based cohort established to investigate incident stroke and related risk factors. Between 2003 and 2007, 30,239 self-reported AA and EA adults aged ≥45 years were enrolled from all 48 contiguous U.S. states and the District of Columbia (Howard et al., 2005). At baseline, participants completed a computer-assisted telephone interview followed by an in-home visit that included blood pressure measurement, phlebotomy, and anthropometrics. REGARDS is ongoing, with participants contacted every 6 months to ascertain incident stroke and secondary cardiovascular outcomes.

Suspected events identified through surveillance were adjudicated through medical record review by trained physicians using the World Health Organization definition: focal neurological deficits lasting >24 h or imaging evidence consistent with acute stroke (Howard et al., 2005; Howard et al., 2011). All ischemic stroke events occurring on or before 30 September 2019, were eligible for inclusion. Participants with a history of stroke at baseline were excluded.

#### The All of Us Research Program

All of Us is a prospective cohort designed to advance precision medicine by recruiting a diverse population across the United States. Enrollment of adults aged ≥18 years began in May 2018 across more than 340 sites. Participants contribute electronic health record (EHR) data, complete baseline demographic and lifestyle surveys, and undergo physical measurements and biospecimen collection (All of Us Research Program Investigators et al., 2019; Ramirez et al., 2022). EHR data include diagnoses, procedures, medications, and laboratory results.

For the present analysis, we used the Controlled Tier version 7 data release (April 2023). The genomic dataset available in version 7 included 245,388 individuals with short-read whole-genome sequencing data (All of Us Research Program Genomics and I, 2024). To harmonize the age distribution with REGARDS, analyses were restricted to participants aged ≥45 years. The reported results comply with the All of Us Data and Statistics Dissemination Policy, which disallows disclosure of group counts <20.

#### The Genetics of Hypertension Associated Treatments (GenHAT) study

GenHAT was an ancillary pharmacogenomics study of the Antihypertensive and Lipid-Lowering Treatment to Prevent Heart Attack Trial (ALLHAT). ALLHAT was a double-blind, randomized clinical trial conducted from 1994 to 2002 that enrolled >42,000 adults aged ≥55 years with hypertension and at least one additional cardiovascular risk factor. Participants were randomized to chlorthalidone, lisinopril, amlodipine, or doxazosin (Davis et al., 1996; The ALLHAT Officers and Coordinators for the ALLHAT Collaborative Research Group, 2000).

The original GenHAT cohort included 39,114 participants (12,520 self-reported AA participants) (Arnett et al., 2002). A subsequent GWAS ancillary study generated genome-wide genotype data on a subset of AA participants randomized to chlorthalidone or lisinopril (Armstrong et al., 2022; Armstrong et al., 2021). Because ischemic and hemorrhagic stroke were not reported separately in ALLHAT/GenHAT, GenHAT analyses used an all-stroke phenotype.

### Stroke endpoint

#### REGARDS

IS events in REGARDS were identified through biannual surveillance and adjudicated via medical record review. For participants aligned to Medicare claims, additional ischemic stroke events were identified from inpatient claims using ICD-9 codes 433. x1 or 434. x1 or ICD-10 codes I63. xx in the primary diagnosis position. Hospitalizations occurring within 1 day of each other were merged into a single episode of care. Participants with self-reported stroke at baseline were excluded.

#### All of Us

To harmonize phenotype definitions with REGARDS, analyses in All of Us were restricted to participants aged ≥45 years. IS cases were defined as individuals with an inpatient encounter associated with ICD-9 codes 433. x1 or 434. x1, or ICD-10 codes I63. xx. Stroke codes identified only in non-inpatient encounters, SNOMED stroke codes, and hemorrhagic stroke codes (ICD-9,430. x, 431. x; ICD-10 I60. x, I61. x) were excluded from both case and control sets.

#### GenHAT

Incident stroke in GenHAT was defined as a persistent neurological deficit resulting from arterial occlusion or rupture, lasting >24 h (unless fatal), or with CT/MRI evidence consistent with acute stroke, excluding stroke due to trauma, infection, tumor, or other non-ischemic causes. Events occurring perioperatively were included (The ALLHAT Officers and Coordinators for the ALLHAT Collaborative Research Group, 2000). In the primary ALLHAT study, the type of stroke (specifically differentiating between ischemic and hemorrhagic) was not systematically classified or reported as part of the trial’s pre-specific secondary outcomes; therefore, stroke was analyzed as an all-stroke phenotype in this cohort.

### Genomic data

#### REGARDS optimization cohort

A subset of 10,551 AA and EA REGARDS participants underwent genotyping using the Illumina Infinium AMR/AFR (MEGA) BeadChip array. Following previously described quality control procedures (Armstrong et al., 2021), internal duplicates and HapMap controls were removed; samples were excluded when genetic sex did not match self-reported sex. Variants were excluded if located on sex chromosomes, were non–biallelic, had ambiguous strand alignment, violated Hardy–Weinberg equilibrium (p < 1.00E-12), had minor allele frequency (MAF) <5%, or had missingness >10%. Genotypes were imputed using the Trans-omics for Precision Medicine (TOPMed) Release 2 (Freeze 8) reference panel (Das et al., 2016).

Ancestry principal components (PCs) were generated using EIGENSTRAT (Price et al., 2006). Primary ancestry groups were defined based on self-reported ancestry, with genetic PCs included as covariates to account for within population substructure. After quality control (QC), 6,342 AA (422 ischemic stroke cases; 5,920 controls) and 1,403 EA (343 ischemic stroke cases; 1,060 controls) were included in the PRS optimization cohort.

### Independent validation cohorts

#### REGARDS (Holdout)

A separate, independent batch of REGARDS genetic data included 12,118 participants of European ancestry genotyped on the Illumina Infinium Global Diversity Array-9 (GDA) with custom content targeting neurodegenerative disease-focused variants (Bandres-Ciga et al., 2024). Variants were excluded if they were located on sex chromosomes, had ambiguous strands, were multi-allelic, were indels, violated Hardy Weinberg Equilibrium (HWE, p < 1.00e-05), and/or had a missing rate >5%. Individuals were excluded based on sex discrepancies (genotyped versus self-reported), internal duplicates, or those with <98% call rates (Armstrong et al., 2026). Genotypes were imputed using the TOPMed release 3 reference panel. PCs were generated using EIGENSTRAT. After QC, 11,255 EA participants (439 ischemic stroke cases and 10,816 controls) remained for validation analysis.

#### All of Us

Short-read whole-genome sequencing (WGS) data from the All of Us Research Program were obtained from the Controlled Tier version 7 release (All of Us Research Program Genomics Investigators, 2024) and analyzed using the program’s standardized genomic pipeline (e.g., variant calling, QC, and imputation). Participants with low sequencing quality, discordant sex, excess heterozygosity, or contamination were removed. Only participants with WGS and complete and relevant phenotype data (n = 107,749; ∼27.5% AA) were included in analyses.

#### GenHAT

Genotyping was performed using the Illumina Infinium Multi-Ethnic AMR/AFR BeadChip (>1.4 M markers), and imputation used the TOPMed Release 2 (Freeze 8) reference panel. Detailed genotyping and imputation quality control procedures have been previously published (Armstrong et al., 2022; Armstrong et al., 2021). Imputation to TOPMed Release 2 (Freeze 8) was performed using Minimac4, retaining variants with imputation quality r^2^ > 0.5. After QC and restriction to participants with available phenotype and genetic data, 6,908 AA GenHAT participants were included in validation analyses.

#### Construction and optimization of IS PRSs

Bayesian PRS methods were constructed using GWAS summary statistics and external LD reference panels. Ancestry-specific GWAS summary statistics were obtained from the GIGASTROKE consortium (Mishra et al., 2022), including results from 1,296,908 EA (GCST90104540) and 20,924 AA (GCST90104550) individuals.

To assess potential sample overlap between the discovery GWAS and validation cohorts, available cohort descriptions from GIGASTROKE consortium studies were reviewed. To our knowledge, REGARDS, All of Us, and GenHAT participants included in the present analyses were not included in the ancestry-specific GIGASTROKE discovery summary statistics that we used for PRS construction. Therefore, all datasets were considered independent of the discovery GWAS.

Prior to PRS calculation, summary statistics and target genotype datasets were harmonized based on chromosome position (hg38) and reference/alternate alleles using the McCarthy Group Tools (Rayner, , 2011). Ambiguous strand variants, allele mismatches, and variants unavailable in the summary statistics, as well as the target genotyped datasets, were excluded during pre-processing. Only variants passing genotype QC and available after harmonization, were retained for PRS calculation.

#### PRS-CS

PRS-CS is a Bayesian polygenic prediction method that estimates posterior SNP effect sizes using GWAS summary statistics and an external LD reference panel (Ge et al., 2019). For PRS-CS analyses, we used the HM3 LD reference panels for EA and AA populations. SNP weights were generated under four values of the global shrinkage parameter (phi, ϕ): 1.0, 1.0E-02, 1.0E-04, and 1.0E-06. PRSs generated using each ϕ were evaluated separately within each ancestry group.

To evaluate whether expanded LD reference panels improve PRS, PRS-CS was additionally applied using the TagIt LD reference panel developed by the D-PRISM Consortium (Huerta-Chagoya et al., 2026). The TagIt panel expands the HM3 SNP set to increase variant density and improve LD estimation across diverse populations (Huerta-Chagoya et al., 2026). As with PRS-CS–HM3, four PRS-CS-TagIt PRSs were generated using the same grid of ϕ values.

#### SBayesRC

SBayesRC is a Bayesian summary-statistics-based method that incorporates GWAS data with functional genomic annotations, including coding, conserved, regulatory, and LD-related features (Zheng et al., 2024). SBayesRC extends SBayesR (Lloyd-Jones et al., 2019) by modeling all imputed common SNPs simultaneously and incorporating annotations through an annotation-dependent multicomponent mixture prior that allows annotations to influence both the probability that a SNP is causal and the distribution of its effect size (Zheng et al., 2024). SBayesRC requires GWAS summary statistics and an LD reference panel as inputs. Heritability and annotation parameters are estimated internally within the Bayesian framework. Outputs include posterior SNP effect estimates for PRS construction, posterior inclusion probabilities, and annotation-specific genetic architecture parameters.

In total, 18 PRSs were generated in the REGARDS optimization cohort (nine per ancestry group): four PRS-CS–HM3 scores, four PRS-CS–TagIt scores, and one SBayesRC score.

#### Statistical analysis

Baseline characteristics of study participants were summarized separately by self-reported ancestry group and cohort. Categorical variables were presented as counts and percentages, and continuous variables were summarized as mean (standard deviation, SD) as appropriate. Statistical analyses were conducted separately within each ancestry group. All PRSs were standardized to a mean of zero and an SD of 1 within ancestry group before analysis. Associations between PRS and incident stroke were evaluated using logistic regression models, with ORs and 95% confidence intervals (CIs) estimated per SD increase in the PRS (OR/SD). Base models (covariate-only) were adjusted for age, sex, and first 10 ancestry PCs. For GenHAT, the antihypertensive treatment randomization group was also included as a covariate due to the randomized trial design of ALLHAT. A PRS only model evaluated the association of IS (or all-stroke in GenHAT) with each individual PRS. A full model was developed for each PRS, evaluating the association between the outcome and PRS, while adjusting for the base model covariates (age, sex, PCs, and randomization group where applicable). All statistical analyses were performed using R version 4.4.2 (R Foundation for Statistical Computing). Two-sided p-values <0.05 were considered statistically significant.

#### Optimization of PRS performance

All PRSs were initially evaluated within the REGARDS optimization cohort to select the best-performing PRS-CS parameterization within each ancestry group. For PRS-CS-HM3 and PRS-CS-TagIt, performance was assessed across four global shrinkage parameters (ϕ = 1.0, 1.0E-02, 1.0E-04, 1.0E-06). Selection of the optimal PRS-CS score was based on multiple performance metrics including the proportion of variation in the trait explained by the PRS on the liability scale (liability-scale R^2^), OR/SD, and area under the receiver operating characteristic curve (AUC), with R^2^ as the primary selection metric. AUCs were calculated for the covariates-only model (age, sex, and PCs), the PRS-only model, and the full model. The optimization cohort was used exclusively for PRS parameter selection and was not considered an independent assessment of predictive performance.

#### Evaluation of PRS performance

The selected PRS-CS-HM3 and PRS-CS-TagIt scores for each ancestry group, along with the SBayesRC score, were subsequently evaluated in independent validation cohorts, including the REGARDS holdout cohort, All of Us, and GenHAT. Primary performance metrics included OR/SD, liability-scale R^2^, and AUC. AUC was evaluated for the covariate-only model, PRS-only model, and full model. Incremental discrimination was assessed using the change in AUC (ΔAUC), calculated as the difference between the full model and the covariate-only model.

The association between PRS and stroke risk was additionally evaluated by comparing individuals in the top 2%, 5%, and 10% of the PRS distribution with the remainder of the population. Sensitivity, specificity, adjusted positive predictive value (PPV), and adjusted negative predictive value (NPV) were estimated using population stroke prevalences of 2.7% and 4.3% for EA and AA, respectively (Imoisili et al., 2024) were used to calculate adjusted PPV, adjusted NPV, and the liability-scale R^2^.

#### Assessment of model calibration and incremental predictive performance

Additional evaluation of predictive performance was conducted in the independent REGARDS holdout and GenHAT validation cohorts. Calibration was assessed using the Brier score for the covariate-only and full models, the change in Brier score (ΔBrier), calibration intercept, and calibration slope, using the R package CalibrationCurves (Van Calster et al., 2016). Calibration curves were generated by comparing observed and predicted stroke risk across deciles of predicted risk and by plotting observed stroke risk against mean predicted risk across deciles of predicted risk for each PRS and ancestry group.

Continuous net reclassification improvement (NRI) quantified the improvement in risk classification associated with addition of the PRS by comparing the proportion of individuals with and without stroke. Integrated discrimination improvement (IDI) was calculated as the difference in discrimination slopes between the full models (covariate + PRS) and covariate-only models, reflecting the improvement in separation of predicted risks between stroke cases and non-cases after addition of the PRS. Confidence intervals for continuous NRI and IDI were estimated using bootstrap resampling (200 iterations) within the REGARDS holdout and GenHAT validation cohorts. To formally compare discrimination across PRS methods, receiver-operating characteristic (ROC) curves were generated using the pROC package in R (Robin et al., 2011), for each PRS-enhanced model within the independent REGARDS holdout and GenHAT validation cohorts. Pairwise comparisons of full model AUCs between correlated ROC curves were performed using DeLong’s test. Comparisons were conducted between PRS approaches within each ancestry group between (i) PRS-CS-HM3 and PRS-CS-TagIt, (ii) PRS-CS-HM3 and SBayesRC, and (iii) PRS-CS-TagIt and SBayesRC to evaluate whether any observed differences in discrimination between PRS methods were statistically significant. P-values were adjusted using the Holm method within each ancestry-specific validation cohort to control the family-wise error rate.

#### Application of external PRS for comparative assessment

We evaluated two publicly available ischemic stroke PRSs retrieved from the Polygenic Score (PGS) Catalog (Lambert et al., 2021; Lambert et al., 2024). The Abraham et al. score (PGS000039) is a metaPRS composed of 19 component scores, and the Mishra et al. score (PGS002724) is an integrative metaPRS comprising 22 component polygenic scores. These published scores were applied to the REGARDS holdout, GenHAT, and All of Us validation cohorts to provide a point of comparison for predictive performance. Because these external scores differ substantially in construction methodology and component variants, direct statistical comparisons with the newly generated PRSs were not performed.

## Results

### Study populations and PRS construction

**FIGURE 1:**
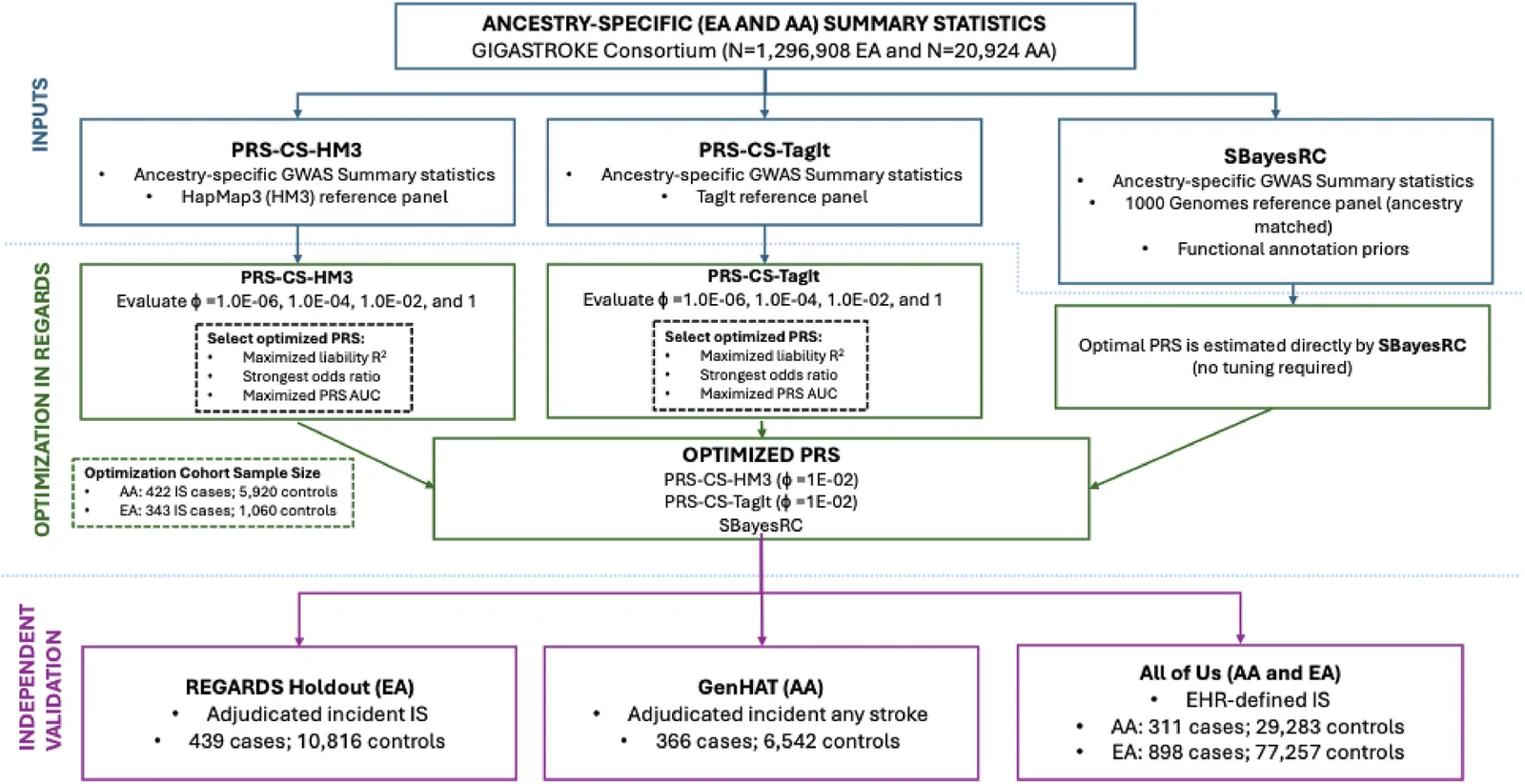
Overview of study design, PRS development and optimization, and validation. Ancestry-specific (EA and AA) summary statistics from the GIGASTROKE consortium were used to construct PRS using three methods: PRS-CS with the HM3 reference panel (PRS-CS-HM3), PRS-CS with the TagIt reference panel (PRS-CS-TagIt), and SBayesRC. For PRS-CS, global shrinkage parameters (phi, ɸ) of 1.0E-06, 1.0E-04, 1.0E-02, and 1 were evaluated, and the parameter that maximized the liability-scale R^2^, had the strongest parameter estimate (odds ratio), and the largest PRS AUC, was selected as the optimized PRS (ɸ = 1.0E-02). For SBayesRC, the optimal PRS is output directly. Optimized PRS were evaluated in three independent cohorts (no participant overlaps with the discovery GWAS used for PRS construction or with the REGARDS optimization cohort). Abbreviations: AA-African ancestry; EA-European ancestry; IS-ischemic stroke; EHR-electronic health records; AUC-area under the curve.

A total of 133,657 participants (42,844 AA) were included across the REGARDS (optimization and holdout sub-cohorts), GenHAT, and All of Us studies after applying quality control filters and restricting analyses to individuals with complete covariate and phenotype data (Figure 1). Participant demographics are summarized in Table 1. The REGARDS optimization cohort included 6,342 AA participants (mean age 63.6 ± 9.2 years; 60.9% female) and 1,403 EA participants (mean age 68.3 ± 10.4 years; 42.3% female) from REGARDS. Independent validation cohorts included 29,594 AA and 78,155 EA participants from All of Us, 6,908 AA participants from GenHAT, and 11,255 EA participants from the REGARDS holdout cohort (mean age range: 63.3–68.8 years; female representation: 51.6%–58.2%).

**TABLE 1:** Sample characteristics of optimization and testing cohorts.

Study	Ancestry	Age, mean ± SD	Female sex, %	N cases	N controls	N total
Optimization						
REGARDS	AA	63.6 ± 9.2	60.9	422	5,920	6,342
REGARDS	EA	68.3 ± 10.4	42.3	343	1,060	1,403
Validation/Testing						
Study	Ancestry	Age, mean ± SD	Female sex, %	N cases	N controls	N total
All of us	AA	63.3 ± 8.1	53.9	311	29,283	29,594
All of us	EA	68.8 ± 10.0	58.2	898	77,257	78,155
GenHAT	AA	66.2 ± 7.8	55.4	366	6,542	6,908
REGARDS holdout	EA	64.0 ± 9.1	51.6	439	10,816	11,255

Abbreviations: AA- african ancestry; EA- european ancestry.

PRSs were generated using three Bayesian approaches: PRS-CS-HM3, PRS-CS-TagIt, and SBayesRC, stratified by ancestry. Variant overlap across validation cohorts was high for all PRS approaches, supporting reproducibility of PRS calculation across datasets (Supplementary Table S1). Among AA participants, PRS-CS-HM3 included 983,900 variants in the optimization cohort (>99% overlap in validation cohorts), PRS-CS-TagIt included 1,368,789 variants (>99.99% overlap in validation cohorts), and SBayesRC included 6,064,174 variants with 89% overlap in All of Us and 98% overlap in GenHAT. Among EA participants, PRSs generated in the optimization cohort included 1,093,847 variants for PRS-CS-HM3 (>98% overlap in the validation cohorts), 1,368,236 variants for PRS-CS-TagIt (>97% overlap in validation cohorts), and 7,356,518 variants for SBayesRC (89% and 97% overlap in All of Us and REGARDS holdout cohort, respectively).

### PRS optimization and performance in independent validation cohorts

PRS parameter optimization was performed exclusively within the REGARDS optimization cohorts, and a ϕ of 1.00E-02 was selected for PRS-CS-HM3 (AA and EA) and PRS-CS-TagIt (AA and EA) (Supplementary Table S2; Supplementary Table S3). Estimates from the optimization cohorts (AA and EA) were not considered independent estimates of predictive performance.

**FIGURE 2:**
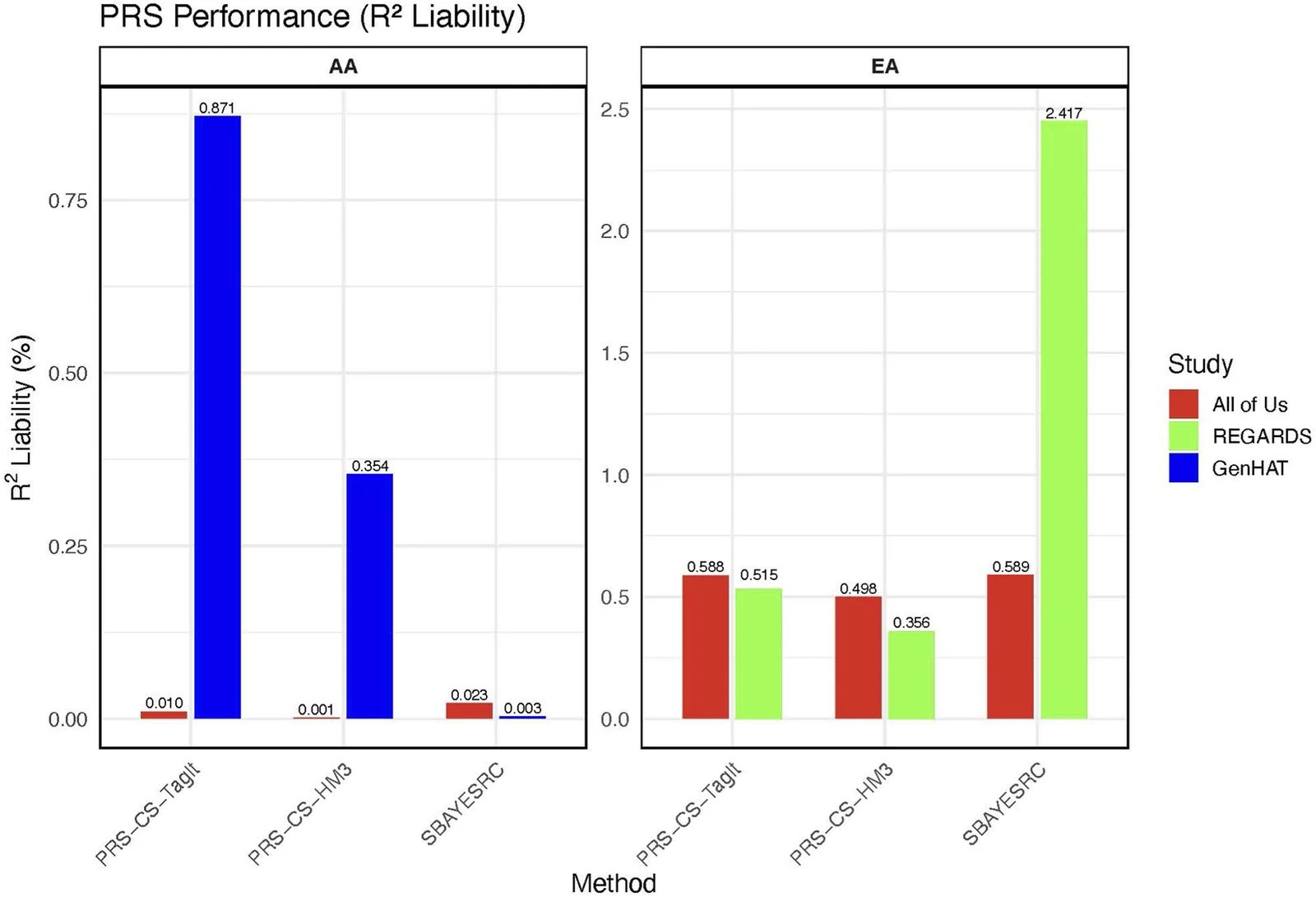
R^2^ liability comparison per 1 SD of PRS among testing/validation cohorts. Liability-scale R^2^ represents the proportion of variance in ischemic stroke risk explained by each PRS per 1 standard deviation (SD) increase in PRS. Results are shown separately for African ancestry (AA, Left Panel) and European ancestry (EA, Right Panel) participants across independent testing/validation cohorts, including All of Us (AA and EA), REGARDS holdout (EA), and GenHAT (AA). PRS performance was evaluated for three Bayesian PRS approaches: PRS-CS using the TagIt linkage disequilibrium (LD) reference panel (PRS-CS-TagIt), PRS-CS using the HapMap3 LD reference panel (PRS-CS-HM3), and SBayesRC incorporating functional annotations. Higher liability-scale R^2^ values indicate greater variance explained by the PRS.

Primary validation results for the selected PRSs are presented in Table 2, Supplementary Table S4, Supplementary Table S5, and Figure 2. Across validation cohorts, associations and predictive metrics were consistently stronger among EA participants compared with AA participants. Among EA participants, SBayesRC demonstrated the largest point estimates for effect size and variance explained among the evaluated PRS approaches. In All of Us participants, the SBayesRC score was associated with incident IS with an OR/SD of 1.16 (95% CI = 1.12–1.19) and R^2^-liability of 0.59%. Similar patterns were observed in the REGARDS holdout validation cohort, where SBayesRC demonstrated an OR/SD of 1.24 (95% CI: 1.12–1.36) and liability R^2^ of 2.42%. PRS-CS-derived scores showed consistent but modest associations across EA validation cohorts (Table 2; Figure 2; Supplementary Table S4).

**TABLE 2:** Validation performance per 1 standard deviation of ischemic stroke PRS.

Ancestry	Study	Method	Or (95% CI)	P	AUC PRS (95% CI)
AA	All of us v7	PRS-CS-HM3^1^	1.04 (0.99, 1.09)	1.30E-01	51.71% (48.08%, 55.34%)
		PRS-CS-TagIt^1^	1.03 (0.98, 1.08)	2.94E-01	49.97% (46.23%, 53.71%)
		SBAYESRC	0.99 (0.94, 1.05)	7.91E-01	50.26% (46.70%, 53.81%)
	GenHAT^2^	PRS-CS-HM3^1^	1.11 (0.98, 1.27)	1.06E-01	51.11% (48.10%, 54.12%)
		PRS-CS-TagIt^1^	1.14 (1.03, 1.26)	9.48E-03	53.68% (50.69%, 56.68%)
		SBAYESRC	1.01 (0.89, 1.14)	8.86E-01	50.15% (46.97%, 53.32%)
EA	All of us v7	PRS-CS-HM3^1^	1.10 (1.07, 1.14)	3.25E-11	53.88% (51.77%, 56.00%)
		PRS-CS-TagIt^1^	1.10 (1.07, 1.13)	8.43E-10	53.85% (51.76%, 55.94%)
		SBAYESRC	1.16 (1.12, 1.19)	1.62E-22	54.47% (52.36%, 56.57%)
	REGARDS (holdout)	PRS-CS-HM3^1^	1.09 (0.98, 1.20)	9.97E-02	52.05% (49.29%, 54.82%)
		PRS-CS-TagIt^1^	1.11 (1.00, 1.22)	4.76E-02	52.18% (49.40%, 54.97%)
		SBAYESRC	1.24 (1.12, 1.36)	1.81E-05	55.16% (52.49%, 57.82%)

1 Optimal phi = 1.00E-02.

2 GenHAT, is evaluating ischemic stroke PRS, on all-stroke phenotype.

Abbreviations: AA- african ancestry; EA- european ancestry; OR-odds, ratio; CI- confidence interval; AUC- area under the curve; HM3- HapMap3.

In contrast, associations and predictive metrics were attenuated among AA participants. In All of Us AA participants, associations for the generated PRSs showed modest associations and were close to the null, with ORs close to 1.0, and R^2^-liability values below 0.1%. In GenHAT, PRS-CS-TagIt provided the strongest association among the novel scores (OR/SD = 1.14, 95% CI: 1.03–1.26, R^2^-liability = 0.87%), although the magnitude of association remained lower than that observed in EA cohorts (Table 2; Figure 2; Supplementary Table S5).

### PRS percentile-based risk stratification

Associations comparing individuals in the highest PRS percentile groups (e.g., 2%–3% strata) with the remainder of the population revealed modest enrichment of stroke events (Supplementary Tables S4,S5). Exploratory thresholds of 3% for EA and 2% for AA were selected based on the distribution of performance metrics observed during PRS optimization in REGARDS.

Among EA participants from All of Us, the top 3% (versus bottom 97%) of the PRS distribution showed increased stroke risk across all PRSs. PRS-CS-TagIt, PRS-CS-HM3, and SBayesRC demonstrated ORs of 1.34 (95% CI: 1.15–1.57, p = 1.52E-04), 1.44 (95% CI: 1.24–1.67, p = 2.65 E−06), and 1.44 (95% CI: 1.24–1.67, p = 2.35E-06), respectively. Similar enrichment was observed in the REGARDS EA holdout cohort, with the strongest associations observed for the PRS-CS-TagIt (OR = 1.71, 95% CI: 1.08–2.71, p = 2.11E-02) and SBayesRC (OR = 1.82, 95% CI: 1.16–2.84, p = 8.80E-03) scores (Supplementary Table S4).

Among AA participants, threshold-based associations (comparing the top 2% of the PRS distribution to the bottom 98%) were less consistent across cohorts. In All of Us AA participants, some nominal associations were observed for PRS-CS-HM3 (OR = 1.49, 95% CI: 1.10–2.02, p = 9.17 × 10^−3^), whereas PRS-CS-TagIt (OR = 1.21, 95% CI 0.88–1.66, p = 2.34E-01) and SBayesRC (OR = 1.20, 95% CI: 0.87–1.66, p = 2.73E-01) were not statistically significant. No PRS demonstrated robust predictive performance in GenHAT (Supplementary Table S5).

Across cohorts, sensitivity values remained limited, ranging from 1% to 25%, while specificity was consistently high (≥90%). Adjusted PPVs were modest, while adjusted NPVs were stable across threshold cut points. Among EA participants, the covariate model achieved AUCs of 66.0% in All of Us and 62.4% in the REGARDS holdout cohort. Addition of PRSs modestly increased AUCs to 66.3%–66.6% for All of Us and 62.6%–63.8% in the REGARDS holdout cohort, depending on the PRS method. Among AA participants, the covariate model achieved AUCs of 66.6% in All of Us and 62.7% in GenHAT, with little or no improvement following inclusion of the PRSs (62.7%–66.7%). Complete per SD or percentile-based estimates for each method, ancestry group, and cohort are provided in Supplementary Tables S4,S5.

### Incremental predictive performance, calibration, and comparison of PRS approaches

Addition of PRS to covariate-adjusted models resulted in modest improvements in discrimination beyond covariates alone. Across validation cohorts, ΔAUC values were small, ranging from 0.002% to 1.33% among EA participants and were smaller than 0.38% among AA participants. The largest improvements in discrimination were observed for SBayesRC in EA participants, although absolute improvements remained modest (Supplementary Tables S4,S5).

Calibration analyses conducted in the REGARDS EA holdout and GenHAT AA validation cohorts demonstrated minimal changes in model performance after addition of PRS. Brier scores showed small absolute improvements for PRS-enhanced models compared to covariate-only models, with the largest reduction observed for SBayesRC in the REGARDS holdout cohort. Calibration slopes were approximately one and calibration intercepts were close to zero across PRS models, suggesting reasonable agreement between predicted and observed risk (Supplementary Table S6). Calibration plots demonstrating observed versus predicted stroke risk across risk deciles are provided in Supplementary Figures S1–S6.

Adding the PRSs to our covariate-only models did not meaningfully improve our ability to classify patient stroke risk. In the REGARDS EA holdout cohort, the SBayesRC method showed the largest improvement, but the actual change in predicted probabilities was negligible (IDI = 0.002, 95% CI 0.001–0.003). All other PRS methods showed similarly minimal improvements over the basic covariates. In the GenHAT cohort, the metrics showed almost no change, and the results were not statistically significant (Supplementary Table S6).

Formal comparisons of PRS discrimination were performed using DeLong’s test comparing ROC curves among PRS-CS-HM3, PRS-CS-TagIt, and SBayesRC. In the REGARDS EA holdout cohort, SBayesRC showed higher discrimination compared to PRS-CS-HM3, although this relationship was not significant when adjusting for multiple comparisons (p_Holm=_ 0.136). Comparisons between PRS-CS-HM3 and PRS-CS-TagIt and PRS-CS-TagIt and SBayesRC were not statistically significant. In GenHAT AA participants, no significant differences in discrimination were observed between PRS approaches (all p > 0.30; Supplementary Table S7). These findings suggest that although some approaches demonstrated numerically improved performance, differences between PRS methods were generally modest. Thus, while expanded LD reference panels and functional annotation-informed approaches showed numerical improvements in some settings, evidence for statistically significant superiority of one PRS method over another was limited.

### Comparison with previously published stroke PRSs

Two previously published ischemic stroke PRSs from the Polygenic Score Catalog (Abraham et al. and Mishra et al.) were evaluated in secondary analyses. Among EA participants, both external PRSs demonstrated positive associations with IS risk, with similar performance between the two scores in All of Us and modest but statistically significant associations in the REGARDS holdout cohort. Addition of the external PRSs resulted in small improvements in discrimination beyond covariate-only models. Among AA participants, associations were weaker and less consistent across cohorts. The Mishra PRS demonstrated modest associations in All of Us AA participants (OR/SD = 1.17, 95% CI: 1.01–1.36) and GenHAT participants (OR/SD = 1.38, 95% CI: 1.19–1.60), although improvements in discrimination remained small, consistent with findings from the newly generated PRSs (Supplementary Table S8).

## Discussion

PRSs have become an increasingly appealing tool in cerebrovascular research, driven by the availability of large-scale GWAS summary statistics and the development of advanced Bayesian modeling approaches. Despite this rapid progress, clinical translation for IS remains limited in comparison to other outcomes (Kurniansyah et al., 2022; Patel et al., 2023), in part because of modest predictive performance and persistent disparities across ancestry groups (Neumann et al., 2021; Moreno-Grau et al., 2024; Abraham et al., 2021). Although methods that incorporate functional annotations or leverage more representative LD reference panels have the potential to improve prediction accuracy and enhance portability across populations (Zeng and Visscher, 2025), their utility for IS PRSs has not been comprehensively evaluated. GWAS have identified hundreds of loci associated with vascular and cerebrovascular diseases, and PRS provide a scalable way to aggregate the polygenic signal (Jagodic et al., 2025). In large consortia studies, IS PRS have shown modest associations that persist after adjustment for major clinical factors, supporting their potential role in early risk stratification (Mishra et al., 2022). However, few studies have systematically evaluated and compared multiple PRS methods for IS prediction in AA adults, despite the disproportionate burden of stroke (Owolabi et al., 2017; Madsen et al., 2024; Howard et al., 2007) in this population. Improving the performance and equitable application of PRS in AA adults is therefore critical to avoid widening existing disparities in genetic risk assessment and future precision prevention strategies. In the present study, we examined whether adding functional annotations and expanding the LD reference panel could improve PRS performance for incident stroke compared with conventional PRS-CS-HM3 approaches among EA and AA adults.

Consistent with prior work suggesting that causal common variants for complex traits are often shared across ancestry groups (Hou et al., 2023), functional annotations have been proposed to help distinguish true causal SNPs from correlated non-causal markers (Li et al., 2024; Weissbrod et al., 2020). However, incorporating annotations increases model complexity and may dilute effect estimates when the number of included variants is large (Zhuang et al., 2024). In our analysis, the annotation-informed PRS (SBayesRC) incorporated approximately three times more SNPs than the standard PRS-CS models. Although functional priors led to a slight improvement in prediction for both EA and AA participants, the magnitude of improvement was modest and insufficient to meaningfully reduce cross-ancestry disparities or outperform previously published scores. One explanation may be that most stroke-associated variants lie in regulatory regions, rather than coding sequences (Malik et al., 2018; Jagodic et al., 2025), limiting the utility of annotation frameworks that prioritize coding or defined functional categories. These results highlight that, while annotation-informed approaches have potential in stroke genomics, careful consideration and model complexity are critical to achieve meaningful improvements in PRS performance across diverse populations.

Prediction performance remained substantially lower among AA participants across all evaluated approaches. This aligns with numerous studies documenting reduced PRS transferability to African ancestry populations (Ju et al., 2022; Martin et al., 2019). The primary drivers are well documented and include underrepresentation in GWAS, differences in LD architecture, and a higher proportion of population-specific variants that are not adequately tagged by current panels or methods (Kachuri et al., 2024). To address this, we evaluated the D-PRISM Consortium’s expanded TagIt LD panel, which includes >1 million additional variants and better represents global genetic diversity. Although PRS-CS-TagIt demonstrated numerically improved performance compared with PRS-CS-HM3 in some analyses, improvements were generally modest especially in AAs and did not translate into consistent statistically significant differences in discrimination based on formal ROC comparisons. These findings suggest that expanded LD reference panels may provide incremental improvements in PRS performance but cannot fully overcome limitations related to ancestry differences in GWAS summary statistic sample size, LD structure, and allele frequency distributions. Notably, our study was focused on the role of functional annotations and updated LD reference information in within ancestry settings. Thus, we did not leverage available multi-ancestry methods such as PRS-CSx (Ruan et al., 2022), Genomic Prediction through Transfer Learning (GPTL) (Wu et al., 2026), BridgePRS (Hoggart, et al., 2024) (Hoggart et al., 2024), or PROSPER (polygenic risk scores based on ensemble of penalized regression models) (Zhang et al., 2024), which should be considered in future IS PRS efforts.

PRS research for IS lags behind other cardiovascular conditions, such as hypertension (Kurniansyah et al., 2022) or coronary artery disease (Patel et al., 2023), where multi-ancestry PRS have been more extensively evaluated and generally show larger effect sizes (often with effect estimates >1.5 per SD (Agbaedeng et al., 2021; King et al., 2022; Mars et al., 2023)). Although our models performed similarly to previously published scores, absolute prediction remained low. This difference likely reflects the complex and heterogeneous biology of stroke, which includes multiple etiologic mechanisms and clinically distinct subtypes. Therefore, even substantial improvements in genetic prediction methodology may yield only incremental gains when applied to IS as a broad phenotype.

Several published stroke PRSs have been constructed as metaPRSs or “mega-scores,” including those developed by Abraham et al. and Mishra et al. Rather than being derived from a single GWAS, these scores integrate multiple trait-specific PRS representing genetically correlated cardiovascular and cardiometabolic phenotypes, such as blood pressure, lipid traits, and atrial fibrillation. This multi-trait architecture allows metaPRS to capture a broader range of biological pathways contributing to ischemic stroke, consistent with the heterogeneous and multifactorial etiology of the disease. Because their construction differs from single-trait or functionally informed PRS, direct comparisons across score types are inherently challenging, and observed differences in predictive performance should be interpreted cautiously. Integrating multiple trait-specific signals may also enhance robustness across cohorts, as the aggregated score is less dependent on any single discovery dataset or genetic architecture. Consistent with this concept, our evaluation of previously published metaPRS approaches demonstrated comparable or modestly improved performance in some settings; however, these scores also did not overcome the broader limitations in cross-ancestry prediction.

A strength of our study is the use of rigorously adjudicated stroke outcomes and harmonized genetic data in a well-characterized U.S. cohort with substantial representation of AA adults for the generation of the PRS. Additionally, the direct comparison of functional priors and LD panel choice within a consistent analytic workflow provides unique insight into the relative contributions of each methodological component. We further evaluated clinical predictive performance beyond traditional association metrics by examining discrimination improvement, calibration, and reclassification measures. Although some approaches improved predictive metrics numerically, the incremental improvement beyond covariates alone was modest, with covariate-only model AUCs ranging from 62.4% to 66.6% and covariate-plus-PRS model AUCs ranging from 62.6% to 66.7% across validation cohorts and PRS methods, highlighting the challenges of translating stroke PRSs into clinically meaningful prediction tools.

However, this study is not without limitations. First, while we relied on single-ancestry summary statistics from the largest-to-date stroke GWAS for both EA and AA populations, our sample size, particularly for the AA analyses, was limited. Larger GWAS efforts, particularly those including greater representation of historically underrepresented populations, will be necessary to improve PRS development and portability. Second, PRS parameter selection for PRS-CS-HM3 and PRS-CS-TagIt was performed within the REGARDS optimization cohort. Although this approach allowed selection of ancestry-specific model parameters prior to independent evaluation, estimates obtained during optimization may be subject to optimism due to model selection. Therefore, optimization cohort results were used only for parameter selection and were not interpreted as independent estimates of predictive performance. Third, we evaluated ancestry-specific rather than multi-ancestry approaches. Although ancestry-specific models allowed direct assessment of population-specific performance, they do not address whether jointly modeling diverse populations may improve PRS portability or predictive performance. Fourth, we focused strictly on IS as a single disease and not subtype-specific IS specifically due to GWAS summary statistic constraints, acknowledging that IS subtypes have distinct genetic architectures and that subtype distributions may differ across ancestries. Fifth, we focused on EA and AA populations due to the composition of REGARDS, which may limit generalizability to other ancestry groups. Sixth, our inability to distinguish ischemic from hemorrhagic stroke somewhat limits the interpretation of the results in GenHAT, however IS known to make up ∼85–90% of stroke cases in the US (Martin et al., 2025); therefore, we felt the inclusion of this data in our validation set was warranted. Finally, while we aimed to harmonize the ischemic stroke phenotype, we note that there is likely heterogeneity in our cases and controls based on the differing study designs (e.g., REGARDS is a population-based longitudinal study with adjudicated outcomes; ALLHAT/GenHAT is a randomized clinical trial with an all-stroke outcome; All of Us through electronic medical records).

In summary, we demonstrate that Bayesian approaches incorporating expanded LD reference panels and functional annotations provided modest improvements in IS performance among EA individuals, but yielded limited gains beyond covariates alone. However, despite these incremental gains, model performance for AA participants remained substantially lower, underscoring persistent ancestry-related disparities. IS risk is shaped by a complex interplay of genetic, clinical, environmental, and social factors, and genetic risk only represents one dimension of this larger framework. As a result, developing PRS that focus on clinical subtypes of IS and then integrating those scores with clinical risk factors, lifestyle patterns, and social determinants will likely be needed to achieve meaningful improvements in prediction. Overall, continued expansion of diverse genomic resources and methodological innovation remains essential to advancing the clinical utility of PRS for stroke prevention and risk stratification.

## Data Availability

The REGARDS (Study Accession: phs002719.v1.p1) and GenHAT (Study Accession: phs002716.v1.p1) phenotypic and genetic data used in this study are available through the National Center for Biotechnology Information (NCBI) database of Genotypes and Phenotypes (dbGaP) to approved researchers following applicable data access procedures. This study also used Controlled Tier data from the All of Us Research Program through an institutional Data Use and Registration Agreement (DURA) which allows UAB researchers who have completed required training, and approvals to access individual level data through the All of Us Researcher Workbench. Individual-level participant data cannot be made publicly available outside the platform. The REGARDS holdout sample used for validation analyses is supported by the National Institute on Aging (NIA) and the National Institute of Neurological Disorders and Stroke (NINDS) through the Center for Alzheimer’s and Related Dementias (CARD) and is made available through the AD Knowledge Portal (https://adknowledgeportal.synapse.org) in accordance with applicable data access policies. Summary statistics from the GIGASTROKE genome-wide association study used for polygenic risk score development are publicly available through the NHGRI-EBI GWAS Catalog (ID: 36180795). Previously published polygenic scores evaluated in this study are publicly available through the Polygenic Score Catalog, including the Abraham et al. score (PGS Catalog ID: PGS000039) and the Mishra et al. score (PGS Catalog ID: PGS002724).The polygenic risk scores developed in this study are found under the publication ID PGP000839 (score IDs PGS019947-PGS019952). Scripts and code for PRS construction are available through GitHub (https://github.com/uabgenepi/stroke_prs/wiki).
