# Supplementary material for: Evaluation of functional annotation-informed and ancestry-specific polygenic risk scores for ischemic stroke
Source: Front Bioinform. 2026 Sep 10;6:1875388. doi: 10.3389/fbinf.2026.1875388 (PMC13601841; doi:10.3389/fbinf.2026.1875388)
Supplement: Supplementary file 1 [file Supplementaryfile1.docx]

**Supplementary Material**

**A.****B.**

**Supplementary Figure S1. Calibration of the PRS-CS-HM3 ischemic stroke prediction model in the GenHAT validation cohort**. Calibration of the PRS-CS-HM3 model was assessed in the GenHAT validation cohort. The model included age, sex, ancestry principal components, baseline antihypertensive randomization group, and the PRS. (A) Observed and predicted stroke risk across deciles of predicted risk. Bars represent the observed proportion of all stroke events, and the mean predicted risk within each decile. (B) Calibration curve comparing observed risk (y-axis) with mean predicted risk (x-axis) across deciles of predicted risk. The dashed diagonal line represents perfect calibration.

**A.** **B.**

**Supplementary Figure S2. Calibration of the PRS-CS-TagIt ischemic stroke prediction model in the GenHAT validation cohort**. Calibration of the PRS-CS-TagIt model was assessed in the GenHAT validation cohort. The model included age, sex, ancestry principal components, baseline antihypertensive randomization group, and the PRS. (A) Observed and predicted stroke risk across deciles of predicted risk. Bars represent the observed proportion of all stroke events, and the mean predicted risk within each decile. (B) Calibration curve comparing observed risk (y-axis) with mean predicted risk (x-axis) across deciles of predicted risk. The dashed diagonal line represents perfect calibration.

**A.****B.**

**Supplementary Figure S3. Calibration of the SBayesRC ischemic stroke prediction model in the GenHAT validation cohort**. Calibration of the SBayesRC functional model was assessed in the GenHAT validation cohort. The model included age, sex, ancestry principal components, baseline antihypertensive randomization group, and the PRS. (A) Observed and predicted stroke risk across deciles of predicted risk. Bars represent the observed proportion of all stroke events, and the mean predicted risk within each decile. (B) Calibration curve comparing observed risk (y-axis) with mean predicted risk (x-axis) across deciles of predicted risk. The dashed diagonal line represents perfect calibration.

**A.** **B.**

**Supplementary Figure S4. Calibration of the PRS-CS-HM3 ischemic stroke prediction model in the REGARDS Holdout validation cohort**. Calibration of the PRS-CS-HM3 model was assessed in the REGARDS Holdout validation cohort. The model included age, sex, ancestry principal components, and the PRS. (A) Observed and predicted ischemic stroke risk across deciles of predicted risk. Bars represent the observed proportion of ischemic stroke events, and the mean predicted risk within each decile. (B) Calibration curve comparing observed risk (y-axis) with mean predicted risk (x-axis) across deciles of predicted risk. The dashed diagonal line represents perfect calibration.

**A.** **B.**

**Supplementary Figure S5. Calibration of the PRS-CS-TagIt ischemic stroke prediction model in the REGARDS Holdout validation cohort**. Calibration of the PRS-CS-TagIt model was assessed in the REGARDS Holdout validation cohort. The model included age, sex, ancestry principal components, and the PRS. (A) Observed and predicted ischemic stroke risk across deciles of predicted risk. Bars represent the observed proportion of ischemic stroke events, and the mean predicted risk within each decile. (B) Calibration curve comparing observed risk (y-axis) with mean predicted risk (x-axis) across deciles of predicted risk. The dashed diagonal line represents perfect calibration.

**A.** **B.**

**Supplementary Figure S6. Calibration of the SBayesRC ischemic stroke prediction model in the REGARDS Holdout validation cohort**. Calibration of the SBayesRC functional model was assessed in the REGARDS Holdout validation cohort. The model included age, sex, ancestry principal components, and the PRS. (A) Observed and predicted ischemic stroke risk across deciles of predicted risk. Bars represent the observed proportion of ischemic stroke events, and the mean predicted risk within each decile. (B) Calibration curve comparing observed risk (y-axis) with mean predicted risk (x-axis) across deciles of predicted risk. The dashed diagonal line represents perfect calibration.

**Supplementary Figure S7. Receiver operating characteristic curves comparing ischemic stroke PRS models for all stroke prediction in GenHAT (AA).**  Receiver operating characteristic (ROC) curves are shown for the covariate (base) model and models incorporating PRS-CS-HM3, PRS-CS-TagIt, or SBayesRC PRSs in the GenHAT validation cohort. The covariate model included age, sex, ancestry principal components, and antihypertensive randomization group. PRS models included the same covariates, with the addition of the corresponding standardized PRS. Areas under the curve (AUCs) are shown in the figure legend.

**Supplementary Figure S8. Receiver operating characteristic curves comparing ischemic stroke PRS models for all stroke prediction in REGARDS Holdout (EA) validation cohort.**  Receiver operating characteristic (ROC) curves are shown for the covariate (base) model and models incorporating PRS-CS-HM3, PRS-CS-TagIt, or SBayesRC PRSs in the GenHAT validation cohort. The covariate model included age, sex, and ancestry principal components. PRS models included the same covariates, with the addition of the corresponding standardized PRS. Areas under the curve (AUCs) are shown in the figure legend.
